# Effect of Seated Trunk Posture on Eye Blink Startle and Subjective Experience: Comparing Flexion, Neutral Upright Posture, and Extension of Spine

**DOI:** 10.1371/journal.pone.0088482

**Published:** 2014-02-07

**Authors:** Erik Ceunen, Jonas Zaman, Johan W. S. Vlaeyen, Wim Dankaerts, Ilse Van Diest

**Affiliations:** 1 Research Group Health Psychology, KU Leuven (University of Leuven), Leuven, Belgium; 2 Department of Clinical Psychological Science, Maastricht University, Maastricht, The Netherlands; 3 Research Group on Musculoskeletal Rehabilitation, KU Leuven (University of Leuven), Leuven, Belgium; University of Münster, Germany

## Abstract

Postures are known to be able to affect emotion and motivation. Much less is known about whether (affective) modulation of eye blink startle occurs following specific postures. The objective of the current study was to explore this. Participants in the present study were requested to assume three different sitting postures: with the spine flexed (slouched), neutral upright, and extended. Each posture was assumed for four minutes, and was followed by the administration of brief self-report questionnaires before proceeding to the next posture. The same series of postures and measures were repeated prior to ending the experiment. Results indicate that, relative to the other postures, the extended sitting posture was associated with an increased startle, was more unpleasant, arousing, had smaller levels of dominance, induced more discomfort, and was perceived as more difficult. The upright and flexed sitting postures differed in the level of self-reported positive affect, but not in eye blink startle amplitudes.

## Introduction

Both dynamic and static body posture are understood to serve a communicative role, as does verbal content, vocal tonality, vocal volume, and facial expression [1]. Darwin [2] already documented that body posture communicates emotional states. This is understood to be a consequence of different emotions having different effects on body posture [3]. Interestingly, the association between emotions and body posture is not merely unidirectional. Several studies indicate that posture also has feedback and regulatory effects on emotion and motivation [4], [5], [6].

The reciprocal influence of posture and emotion may have relevant implications for well-being and for emotion research. It is plausible that the emotional well-being of office workers worldwide is affected by frequently sitting in a slouched posture for extended periods of time given the documented effects of such a posture [7], potentially resulting in more than just back pain. Apart from well-being, emotion research may ought to control for the posture of participants, even if posture is not the main variable of interest. Indeed, posture is potentially a confounding variable; it can affect outcomes of similar studies on emotion differently [8]. As such, the body posture-emotion association is an avenue of research that needs further investigation.

Research on how emotion is affected by posture has primarily been investigated via self-report [5], [6] and behavioral task performance [4], but need not be limited to these measures. One well-established physiological measure of emotion is affective modulation of eye-blink startle [9]. The eye blink startle reflex consists of the activation of the orbicularis oculi muscle surrounding the eye in response to a startling stimulus. This is usually a short burst of white noise and is referred to as an auditory startle probe. The magnitude of the startle response is modulated by emotional valence, and is considered a well-established psychophysiological measure capable of distinguishing between the approach-avoidance dichotomy of emotions [10], [11]. With presentation of pleasant stimuli triggering approach motivation, the startle magnitude in response to an auditory startle probe is reduced relative to a neutral emotional state, whereas it is increased when aversive stimuli related to avoidance motivation are presented. Although this emotional modulation is a robust finding in response to a varied range of emotional stimuli [9], the effect of posture on emotional modulation of startle has received scant attention thus far.

At the time of writing, we know of only one published research paper addressing modulation of startle in relation to posture [12]. It reports on a study that examined the effect of posture on startle during exposure to pictures high in approach motivation versus neutral pictures, matched for content. Results indicated that leaning forward – the posture most congruent with the approach motivation pictures [13]– increased the relative inhibition of startle magnitude in response to approach related pictures more so than did a reclining posture.

One other, unpublished study by Wielgosz and colleagues [14] demonstrated an interaction between posture and presence of threat. Assuming a ‘protective’ posture (i.e., shoulders shrugged) in a context with threat of mild electric shocks helped to decrease startle magnitude relative to assuming an open posture (i.e., shoulders drawn back) in the same threatening context. In the threat-free context, however, the protective posture elicited an increase in startle magnitude relative to startle elicited during an open posture in that same threat-free context. Additionally, the outcomes indicated that increased effort associated with holding either a protective or open posture for several minutes led to increased startle magnitude relative to magnitudes measured during the minutes in which a more effortless neutral upright posture was held, regardless of context.

It is interesting to note that Price, Dieckman and Harmon-Jones [12] classified postures based on approach motivation and compared the difference between inclining and reclining, whereas Wielgosz and colleagues [14] classified postures based on anticipation of threat by comparing the use of the shoulders in protecting versus exposing oneself. Inclination of the upper body versus reclination, and shoulder positioning are obviously not the only variables in posture that relate to emotion. The sagittal position of the spine (in coordination with position of head and shoulders) is another postural variable frequently associated with emotion. Specifically, flexion of the spine and protraction of head and shoulders (i.e., slumping/slouching) is associated with unpleasant, sad emotional states, whereas an upright posture, but with extension of the upper spine and retraction of head and shoulders (arching the back, sticking out the chest) is associated with positive affect, pride and/or an (over)confident state of mind [2], [15], [16]. In particular, the contrasting emotions associated with flexion versus extension of the spine, makes variation of spinal posture an interesting variable for research on the effect of posture on emotion. Although no research has previously been conducted on startle modulation in response to manipulation of spinal posture in the sagittal plane, the idea for the current study did not arise in a vacuum. Contrasting flexion and extension of the spine and observing their effect on startle was inspired by previous findings on startle in relation to unpleasant bodily sensations. Whereas unpleasant gastric stimulation appears to be associated with startle potentiation [17], increasing evidence suggests that dyspneic stimulation is not [18], [19], [20]. We hypothesized that the contrasting findings with the unpleasant bodily stimulations could be due to spinal posture associated with these two different types of stimulation, or with associated tension in muscles that regulate spinal posture. Whereas dyspnea is associated with spine extension [21], stomach ache instinctively leads to spine flexion, a posture associated with easing of gastro-intestinal function [22]. The hypothesis that posture could be responsible for the difference in results in these two types of bodily stimuli was reinforced by the notion that body posture affects emotion (as discussed above), which in turn is known to modulate startle. Additionally, the notion that startle is associated with flexion of the spine in the whole body startle [23], [24] may imply that posture prior to and during startle has the potential to modulate not only the whole body startle as observed earlier [25], but also the eye blink startle magnitude.

To investigate this hypothesis, we set up the current study. The main aim was to explore whether different spinal sitting postures affect self-reported emotion and eye blink startle differently, with the postures under investigation being a flexed, a neutral upright, and an extended spine. As discussed earlier, the effort associated with postures can affect startle magnitude regardless of the specific postural manipulation [14]. As different upright sitting postures are associated with different trunk muscle activation patterns [26], the effort associated with each of the three postures in our study may vary. To account for any relation between startle response and effort in assuming each posture, we also included questions on discomfort experienced, and difficulty maintaining each specific posture, both reflecting emotional correlates of effort.

## Materials and Methods

### 1. Participants

Thirty-six psychology freshmen (mean age = 19.44 years, range 18–30 years, 29 women) participated in return for course credit. Exclusion criteria were pain-related conditions (lower back pain, stomach ache, or others), known or obvious abnormal kyphosis, lordosis, or scoliosis, presence or history of psychiatric disorders and/or epilepsy, and current usage of psychopharmacological agents.

### 2. Ethics Statement

Prior to participation, all subjects read and signed an informed consent: the consent guaranteed anonymity, and stated that participation was voluntary and could be terminated at any point in time without loss of the promised course credit. The study had been approved by both the Psychological and Medical Ethical Committees of the University of Leuven, Belgium and was in accordance with the Declaration of Helsinki [27].

### 3. Sitting Postures

**Figure 1:**
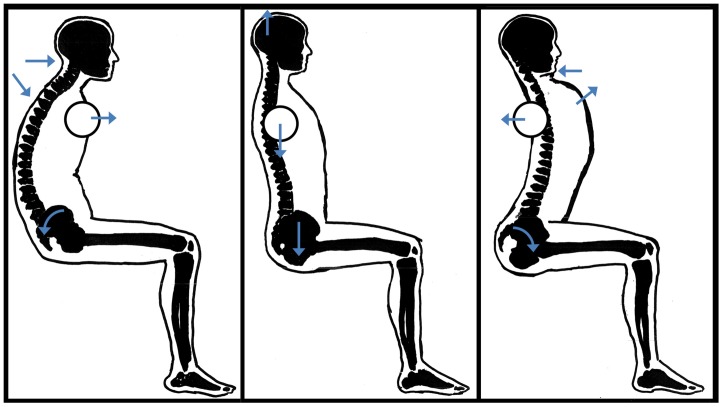
Postural manipulations. These illustrations accompanied the verbal instructions for the flexed (slouched), neutral upright, and extended posture, displayed here respectively from left to right. Negatives of these three illustrations (black background, white figures) were shown one at a time. Arrows appeared one by one during verbal instruction to highlight the four essential aspects that had to be respected.

Each sitting posture had four essential aspects that had to be respected. These were: (1) the position of the pelvis, (2) the position of the upper back, (3) the position of the head, and (4) the position of the shoulders. (See *Figure 1*) Although we have named the postural manipulations by their effect on the spine (flexed, upright, extended), take note that the postures include all four of the listed aspects, i.e. including head and shoulder positioning. Though these two postural aspects may not appear to be direct manipulations of spinal posture, head and shoulder position in fact do contribute significantly to the ability to manipulate the spinal posture of respectively the cervical and thoracic regions of the spine as desired. The postures associated with the dyspnea and stomach discomfort, as well as the whole body startle posture described in the introduction, were seminal in the creation of the postures under investigation in the current study.

#### 3.1. Flexed posture

In order to have the spine in a flexed position, participants were asked to perform a posterior pelvic tilt, and to curve the upper back into maximal kyphosis. Additionally, both the head and the shoulders had to be protracted. Participants were instructed to assume this posture without exerting the abdominal muscles needlessly, as each posture had to be held for four minutes on end.

#### 3.2. Upright posture

In order to have participants assume a neutral upright sitting posture with normal curvature of the spine, participants were asked to position their pelvis neutrally by sitting straight on their sitting bones, and by ‘pulling’ their head upward from the crown. Shoulders were held next to the body in a relaxed (as opposed to shrugged) position. Attention was paid that the upper back was neither slouched forward (kyphotic), nor curved backward (hyperextended).

#### 3.3. Extended posture

To sit in an extended spinal posture, subjects performed an anterior pelvic tilt, curved their upper back in a posterior direction, and retracted head and shoulders.

Note that the postural manipulations in the current study were not primarily intended to imitate displays of negative and positive emotional states, and therefore differ from these emotional displays in certain respects. For example, pride involves outward expansion of the chest [2] which gives it some visual likeness to the extension posture in the current study. In contrast to the expression of pride, the ‘extension’ posture in the current study includes an additional anterior pelvic tilt, which is not seen in the ‘more upright’ posture associated with pride [2]. As such, the extended posture is dissimilar from that of pride, especially on a proprioceptive level. For this reason, individuals assuming the extended posture in our study may not at all have the typical associations with the positive emotion of pride and the accompanying high dominance [28], nor the enhancement of motivational responses [4].

#### 3.4. Manipulation check

The experimenter, although in another room, was able to monitor the participants’ overt compliance with the instructions by means of a closed-circuit video monitoring system. Additionally, three blinded observers retrospectively conducted a forced choice task of classifying still shots of all six postures (3×2) of each participant. These still shots were extracted from a video-recording device which was positioned laterally on the left hand side of the participant. To ensure anonymity of participants, a black oval was inserted on the profile view of their face while leaving the backside of the head visible in order to allow the blinded observers sufficient detail to score the postures correctly.

### 4. Self-report Measures

At the end of each posture a computerized 9-point scale of the language-free Self-Assessment Manikin [SAM-Scale, 29] and computerized Borg scales were administered. On the SAM the subjects had to retrospectively rate the mean valence (unpleasant = 1; pleasant = 9), arousal (calm = 1; excited = 9), and dominance (lack of control = 1, sense of control = 9) they had experienced while adopting the specific posture they had most recently assumed. Borg Scales for perceived mean discomfort and mean difficulty during the posture ranged from 0 to 10 and were labeled from none (0) to maximal (10). After each posture and after filling in the computerized SAM and Borg scales, subjects had to answer a paper and pen version of the Dutch version of the Positive And Negative Affect Schedule (PANAS)-state questionnaire [30].

### 5. Somatic Reflex Measurement and Processing

#### 5.1. Eye blink startle response

Eye blink startle responses were elicited by binaural acoustic presentations of short bursts (50 ms) of white noise (95 dB). Two electrodes filled with high conductivity Microlyte electrolyte gel measured the electromyographic (EMG) activity of the left orbicularis oculi muscle as a response to the acoustic startle probes at the sites specified by Blumenthal et al. [31]; a ground electrode was placed on the center of the forehead. To reduce inter-electrode resistance, all sites were first cleaned with alcohol. The EMG signal was amplified by a Coulbourn isolated bioamplifier (LabLinc v75-04) with a 13 HZ high pass, and 1 KHz low pass bandpass filter. This signal was then routed to a Coulbourn integrator (LabLinc v76-24), which rectified and smoothed the signal with a time constant of 20 ms. The startle EMG was sampled at 1000Hz and recorded starting from 500 ms prior to probe onset, until 1000 ms after probe onset. Although impedance values after attachment of electrodes have not been measured, the appearance of spontaneous blinks during the monitoring of the startle EMG signal confirms that resistance was in normal ranges and reduces the likelihood that there were drifts in impedance affecting the signal.

#### 5.2. Software

A 16-Bit National Instruments PCI-6221 data acquisition card (National Instruments, Austin, Texas) transmitted the EMG signals from the Coulbourn modules to a computer. Affect 4.0 software [32] was used for timing the presentation of startle probes as well as for data acquisition. A program named PSychoPHysiological Analysis, abbreviated as PSPHA [33] was used to handle the recorded signals offline and to extract the relevant parameters necessary for statistical analysis.

### 6. Procedure

We created six groups, as six orders of presentation were possible based on the three postures (the orders were FUE, FEU, EUF, EFU, UEF, and UFE, with F = flexed, U = upright, and E = extended). Participants were randomly assigned to one of these groups, with the constraint that there were equal numbers of participants assigned to each group. An attempt was made to keep the ratio of men for all six posture orders approximately equal, with one male participant per group in five out of six groups, and two males in a sixth group. Upon arrival, the experimenter provided participants an informed consent, which they were requested to read and sign. Next, EMG electrodes were attached – subjects were informed that these were meant for measuring physiological responses, albeit without further specifications. The experimenter then verbally went through the experimental procedure, assisted by on-screen, step-by-step depictions of each of the postures and their essential aspects, and also each of the computerized self-report scales. Additionally to onscreen depictions, essential aspects for each posture were demonstrated by the experimenter (pelvic ∼, upper back ∼, head ∼, and shoulder positions), followed by a request to the participant to briefly assume the posture. The latter was done in order for the experimenter to assess whether participants were able to correctly assume the desired postures. Participants were told that the computer monitor would display when to assume which particular posture. The time for each posture started ticking only after subjects had assumed the posture. Participants were also told to keep their gaze in the direction of the computer monitor on which a fixation cross would appear throughout the experiment. If participants indicated they had no further questions, headphones were placed on their ears, and the experimenter left to the adjacent operator room. Lights remained on (not dimmed) throughout the entire experiment.

The experiment started with a habituation phase in which 10 startle probes were administered to reduce the effect of novelty of startle probe on startle magnitude [31]. After this habituation phase, startle probes were presented on average every thirty seconds during a posture, although the exact time of administration was kept variable. While keeping their gaze at a fixation cross on a computer screen, each of the three postures was assumed for four minutes, with eight startle probes delivered per posture. Once a minute, shortly after administration of a startle probe, a picture of the posture the participant was expected to continue assuming, appeared on the computer screen to remind the participant of each of the essential aspects (pelvic ∼, upper back ∼, head ∼, and shoulder positions) indicated by arrows embedded in the picture. After each posture and before continuing to assume the next posture, participants rated the aforementioned self-report scales. Once all three postures were assumed a first time, the same three postures were repeated a second time in exactly the same order of presentation, while again rating all self-report questions after each posture.

### 7. Data Analysis

Eye blink startle data and Self-report data of this study are publically available, and can be retrieved via http://dx.doi.org/10.6084/m9.figshare.865659.

#### 7.1. Manipulation check

The labels given by each of the three raters were checked on the percentage of postures misidentified. We also checked if the postures of specific participants were misidentified by more than one rater. An inter rater reliability analysis using the Fleiss Kappa statistic was performed to determine consistency among raters in indentifying the three postures.

#### 7.2. Eye blink startle response

Eye blink startle EMG responses were calculated by subtracting the mean baseline value (0 to 20 ms after probe onset) from the peak value found in the 21 to 175 ms time window after probe onset. Startles measured during habituation were excluded from data analysis. EMG measures were visually inspected for presence of spontaneous blinks or other phasic muscular tension of the orbicularis oculi muscle present at the onset of the startle probe and rejected if necessary. As a result, three participants (1 male, 2 females) were excluded since 30% or more of their startles were rejected. Because we were interested in intra-individual differences in response amplitude and not in inter-individual differences, startle probes were transformed to T-scores [31]. Mean startle amplitudes were calculated for each posture per participant per series. Analysis was performed using SPSS 20. Linear mixed models analysis was performed. In order to test the effects of posture, two dummy variables were created, one coding for the flexed (D_Flexed 0/1) and one for extended posture (D_Extended 0/1). The upright posture served as reference (reference coding) [34]. The model had mean startle T-scores as criterion variable and as continuous predictors Valence, Arousal, Dominance, Discomfort, Difficulty, Negative affect and Positive affect; as categorical predictors Series (1/2), Flexed posture (0/1) and Extended posture (0/1) were included. All continuous predictors were centered around the person’s mean [34], [35]. A repeated measures random effects defined by a Series*Position interaction was included. Compound Symmetry was preferred over Unstructured as covariance structure (X^2^_19_ = 27.018, p = .104) as an increase in model complexity did not result in a significant better fit). For the regression parameter estimates, unstandardized coefficients (B’s) are reported and Cohen’s *d* are displayed in Table 1. Cohen’s *d* values larger than.2, .5 and .8 are respectively described as small, medium and large effect sizes (Cohen, 1988).

**Table 1:** The estimates (B), standard errors (SE), flagged significances and Cohen’s d for the multiple regression with reference (dummy) coding.

	*B*	*SE*	*d*
**Intercept**	51.859***	1.026	*7.458*
**Valence**	−.003	.376	*–*
**Discomfort**	−.322	.336	*–*
**Difficulty**	−.742*	.312	.*37*
**Arousal**	.150	.268	*–*
**Positive affect**	. −.181	.896	*–*
**Dominance**	−.261	.263	*–*
**Negative affect**	6.217***	.1.513	.*64*
**Series**	−2.135***	.632	.*526*
**D_Flexed**	.746	.722	*–*
**D_Extended**	3.236***	. 951	.*53*

*Note.* Unstandardized coefficients (B’s) are reported. Effect sizes of parameter estimates are reported as Cohen’s *d*. ***: p≤.001, **: p≤.01, *: p≤.05.

#### 7.3. Self-report

Analysis of the effect of posture on the self-report measures was done using STATISTICA 10. An α-level of.05 was set for statistical significance and partial squared éta effect sizes (η_p_^2^) are reported. Greenhouse-Geisser corrections for violation of sphericity were applied when appropriate. Excluded participants from the startle analysis (see Section 2.6.1.) were also omitted from the self-report analysis. The measures of perceived valence, arousal, dominance, discomfort and difficulty were all separately entered into a 3×2 repeated measures ANOVA with POSTURE and SERIES as within subject variables. Of the PANAS-state, the Positive Affectivity (PA) score was analyzed separately from the Negative Affectivity (NA) score. Significant effects on any of the self-report items were further subjected to Tukey-Kramer post-hoc testing.

## Results

### 1. Manipulation Check

One observer had misidentified only 2% of all postures, while the other two observers each had misidentified only 1% of all postures. There was no overlap between observers on misidentifications: that is, any postures that were misidentified were only misidentified by one observer. Flexed postures were never misidentified. The inter rater reliability was found to be Kappa = 0.96 (p<0.001), 95% CI (92.19, 98.24).

### 2. Eye Blink Startle

The model significantly predicted startle amplitudes (chi^2^_10_ = 60.719, *p*<.001) compared to the most parsimonious model (no predictors, only intercept). There were no effects of Valence, Arousal, Discomfort, Dominance and Positive affect. There was a significant effect of Difficulty (*B* = −.742, *t*(165) = 2.3815, *p* = .018), suggesting that the more a posture was perceived as difficult the lower startle amplitudes were. There was a strong effect of Negative affect (*B* = 6.217, *t*(165) = 4.110, *p*<.001); the higher Negative affect scores were, the higher startle amplitudes were. Startle amplitudes habituated over time, as indicated by the negative beta of Series (*B* = −2.135, *t*(165) = 3.380, *p* = .001). As expected, there was an effect of posture on startle amplitudes. During an extended posture, startle amplitudes were significantly higher compared to an upright posture (*B* = 3.236., *t*(165) = 3.402, *p* = .001) and a flexed posture (*B* = 2.502, *t*(164) = 2.845, *p* = .005), while the flexed posture did not differ from the upright posture (*B* = .746, *t*(165) = .1.032, *p* = .304). This was analyzed through an identical model apart from the dummy variable (D_Flexed), which was replaced by a dummy variable for an upright posture (D_Upright), so that the flexed posture served as reference. See table 1 for details. Stability of the full model and the independent contributions of predictors were confirmed by examining separate simple effect models and model comparisons between models with posture or self-report predictors and their combination.

### 3. Self-report

Repeated measures ANOVAs with POSTURE and SERIES as within subject variables, indicated a main effect of POSTURE for valence, *F*(2, 64) = 32.17, *p*<.001, η_p_^2^ = .50, arousal, *F*(2, 64) = 9.91, *p*<.001, η_p_^2^ = .24, dominance, *F*(2, 64) = 9.57, *p*<.001, η_p_^2^ = .23, discomfort, *F*(2, 64) = 35.48, *p*<.001, η_p_^2^ = .53, difficulty, *F*(2, 64) = 36.48, *p*<.001, η_p_^2^ = .53, and both the PA, *F*(2, 64) = 4.32, *p* = .02, η_p_^2^ = .12 and NA items of the state PANAS, *F*(2, 64) = 4.44, *p* = .02, η_p_^2^ = .12. Further Tukey-Kramer post-hoc testing indicated that the extended posture was significantly more unpleasant, more arousing, had smaller levels of dominance, induced more discomfort, and was perceived as more difficult (for all items, *p*<.002) than both of the other postures which were never significantly different from one another. Regarding the PANAS, post-hoc tests indicated that an upright posture was associated with significantly more PA than a flexed posture (*p* = 0.02). (The level of PA associated with the extended posture did not significantly differ from either that of the upright or flexed posture). NA was significantly higher in the extended posture than in the flexed posture (*p* = 0.01), but NA during the upright posture was not significantly different from either extended or flexed posture. See table 2 for details.

**Table 2:** Means and standard deviations for overall valence, arousal, dominance, discomfort, difficulty, Positive Affect (PA) and Negative Affect (NA) experienced during each of the three postures.

	Flexed	Upright	Extended
SAM – Valence (1 = unpleasant, 9 = pleasant)	4.45^a^ (1.77)	5.12^a^ (1.65)	2.56^b^ (1.04)
SAM – Arousal (1 = calm, 9 = aroused)	3.36^a^ (1.86)	3.67^a^ (1.66)	4.74^b^ (1.89)
SAM – Dominance (1 = not dominant, 9 = dominant)	5.17^a^ (1.79)	5.52^a^ (1.60)	4.24^b^ (1.79)
Borg – Discomfort (0 = none, 10 = maximal)	3.33^a^ (1.77)	2.82^a^ (1.49)	6.02^b^ (2.16)
Borg – Difficulty (0 = none, 10 = maximal)	2.97^a^ (1.66)	2.55^a^ (1.70)	5.38^b^ (2.05)
PANAS – PA (1 = very little, 5 = a lot)	2.17^a^ (0.73)	2.3^b^ (0.71)	2.21^ab^ (0.72)
PANAS – NA (1 = very little, 5 = a lot)	1.26^a^ (0.34)	1.31^ab^(0.43)	1.37^b^(0.39)

*Note.* SAM values of 5 are considered everyday baseline levels of respectively valence, arousal and dominance. Means in the same row which share a subscript are not significantly different from one another according to Tukey-Kramer post-hoc tests. Standard deviations are indicated by the numbers between brackets.

There was also a main effect of SERIES for difficulty, *F*(1, 32) = 8.01, *p* = .008, η_p_^2^ = .20, with the second series of postures being perceived as more difficult than the first. A main effect of SERIES was also present for PA, *F*(1, 32) = 14.72, *p*<.001, η_p_^2^ = .32 of the PANAS, with less PA during the second series. No other main or interaction effects were found.

## Discussion

The current study was an exploration of the effects of spinal posture on subjective experience and eye-blink startle. So far, only one prior publication [12] and one unpublished study [14] included startle as a primary dependent variable in research on the bottom-up effects of posture on emotion. These studies respectively manipulated inclination versus reclination of the upper body, and shoulder positioning. To our best knowledge, our study was the first to systematically manipulate the spinal posture on a sagittal plane during sitting. It included flexion and extension of the spine, as well as an additional upright posture with neutral spinal curvature. All three postures were held for four minutes each, and then repeated a second time. A blinded manipulation check suggests all participants assumed the postures correctly. That the accuracy of identification was slightly less than 100% is presumably due to a combination of clothing and camera angle masking the extended curvature of the back, thereby reducing visual differences between upright and extended postures on the images.

Using these postural manipulations, we found that the extended posture was associated with significantly increased subjective unpleasantness, arousal, discomfort, and difficulty, and a decreased level of dominance relative to both other postures. The extended posture was also characterized by increased state NA relative to the flexed posture. Other than the extended posture, the upright posture was associated with higher state PA relative to the flexed posture. Repeating the series of all three postures a second time led to a decrease in PA, and an increase in perceived difficulty.

As for startle, the extended posture was associated with an increase in startle amplitude relative to both other postures. Increased state NA also led to increased startle, regardless of posture. Increased difficulty paradoxically led to a decrease in startle when all other factors were held constant, even though the extended posture had both the highest mean startle and highest mean difficulty. That eye blink startle amplitude is smaller when difficulty increases, appears to be contrary to the well-documented increase in startle magnitude that is typical for unpleasant emotional states [9]. On the one hand, this could be an indication that difficulty is not necessarily related to negative affect. On the other hand, we need to take into account that reduction in startle amplitude is not necessarily indicative of an absence of unpleasant affect. Several studies suggest that orientation of attention to bodily sensations reduces responsivity to an auditory startle eliciting probe [36], [37], [38]. Sensations of muscle tension and effort associated with postures perceived as difficult, may shift attention to bodily sensations and can as such be responsible for the observed reduction in startle responsivity with difficult postures – even if such postures induce unpleasant affect. Further, a note of caution is in order, as there is a significant possibility of Type II error taking the posture and self-report findings together, as has been done here for the startle analysis.

From the self-report data, the affective changes occurring in conjunction with the extended posture are suspected to be predominantly due to the unpleasant effort associated with it, and not due to pre-existing associations with that body posture. If the affective changes were due to the meaning associated with the body posture, then the flexed posture should be standing out as most negative [7], not the extended posture. The assumed extended posture is a rather unusual sitting posture, especially considering that ‘sticking the chest out’ was not performed in isolation, but in combination with an anterior pelvic tilt. This makes it different from the expression of pride, where the chest is slightly expanded outward without tilting the pelvis [2]. Because the inclusion of the anterior pelvic tilt in this posture, the resulting posture is not one used to express emotions, therefore any negative affect resulting from such a posture is unlikely to be due to associations with that particular body posture. Rather, any negative affect here is most probably due to the muscular effort needed to assume and maintain that posture.

Our data do provide some evidence that pre-existing emotional body posture associations may also exert an effect on affective state when assuming a body posture. In support of this, we like to point out that PA scores were significantly lower during the flexed, i.e. slouched posture as compared to sitting upright. This finding can be interpreted as an indicator of a pre-existing association of PA with sitting upright in our participants [2], and the absence thereof when sitting in a slouched position.

These conclusions as inferred from our data suggest avenues for future research. Our study suggests that any uncomfortable, inexpressive posture will evoke higher startles and more unpleasantness than postures with a pre-existing association with an aversive, negative emotion. This is an assumption that can be tested relatively easily after identifying other uncomfortable, inexpressive postures. These postures can then be contrasted to postures used in expressing negative emotions. Given that uncommon, inexpressive postures require activation of muscles that are relatively untrained, such postures may be suspected to induce an unpleasant affective state by eliciting muscle soreness and perhaps some level of discomfort or pain. For this reason, we advise that future studies evaluating startle reflex include post hoc questions on whether pain was experienced during the posture, and if so, to which extent.

In future studies, it would be of additional interest to find a physiological correlate that is able to measure the effect of postures on emotion, which are due to pre-existing body posture associations, rather than due to effort. Our study suggests that PA remains relatively unaffected by unpleasant effort, and is likely the result of pre-existing emotional associations with specific postures. One method for detecting PA physiologically regardless of arousal is by measuring the post-auricular reflex [39]. Including this measure in future research may be more fruitful in paradigms that are primarily concerned with the effect of different body postures on emotion that are not due to effort, but due to pre-existing body posture associations.

Further implications of our findings are that future studies aimed at pinpointing the effect of embodiment on emotion, particularly on negative emotions, should try to devise postural manipulations that keep the required effort associated with the different postures equal and as minimal as possible. A more general implications is that future studies on emotion with no particular focus on postural manipulations, should at all costs avoid positioning their subjects in an effortful posture in order to limit confounding.

In conclusion, our findings underscore that posture, and especially the effort associated with adopting a specific posture affects both the affective state and eye blink startle magnitude of individuals. We hope that emotion researchers take note that any strenuous posture may affect their results thus should be avoided, unless a strenuous posture is the manipulation under investigation. If emotion is the subject of the study and a strenuous posture cannot be avoided, then care needs to be taken in interpreting the results. As up to now inclusion of psychophysiological measures such as startle in research on the effects of posture is relatively scarce, we consider our conclusions to be preliminary and in need of further testing, replication and extension using the same and other psychophysiological measures of emotion, as well as a variety of postural manipulations.
